# ﻿Description of a new species of *Parens* Fibiger, 2011 (Lepidoptera, Erebidae, Hypenodinae) from Korea

**DOI:** 10.3897/zookeys.1193.113303

**Published:** 2024-02-28

**Authors:** Ji-Young Lee, Bong-Kyu Byun

**Affiliations:** 1 Department of Biological Science and Biotechnology, Hannam University, Daejeon, 34054, Republic of Korea Hannam University Daejeon Republic of Korea

**Keywords:** Checklist, identification key, Micronoctuini, new species, taxonomy

## Abstract

The genus *Parens* comprises small moths, with a wingspan of 9–13 mm, belonging to the family Erebidae. Until now, only four species have been described worldwide. In Korea, only one species, *P.occi* (Fibiger & Kononenko, 2008) has been known to date. In this study, a new species from Korea, *P.fibigerina* Lee & Byun, **sp. nov.**, is described. As a result, two *Parens* species are now known from Korea. Figures of adults, male and female genitalia, and a key to the species of *Parens* in Korea are provided.

## ﻿Introduction

The genus *Parens* Fibiger, 2011 belongs to the tribe Micronoctuini, subfamily Hypenodinae of the family Erebidae. Early authors considered *Parens* as a member of the family Micronoctuidae based the simple male genitalia without a uncus and the two-veined hindwing (Kononenko and Han 2007; Fibiger and Kononenko 2008; Fibiger et al. 2011; Fibiger 2011). More recently, Zahiri et al. (2012) proposed a new taxonomy of the Erebidae based on molecular phylogenetics. The family Micronoctuidae was downgraded to a tribe within the subfamily Hypenodinae, with its subfamilies given the rank of subtribes. This genus was established by Fibiger (2011) with *Parensparaocci* Fibiger, 2011 as the type species. *Parens* is a relatively small genus with only four recognized species worldwide. In Korea, only one species, *P.occi* (Fibiger & Kononenko, 2008) is known, which was first reported by Kononenko and Han (2007) as *Micronoctua* sp. In the present study, a new species, *P.fibigerina* Lee & Byun, sp. nov., is described from Korea.

## ﻿Materials and methods

### ﻿Terminology

We follow the general terminology proposed by Fibiger (2011).

### ﻿Collection and preparation of specimens

The specimens were mainly collected using a bucket light trap with a 20 W black-light lamp and a LepiLED, standard model (WIF, Dr Gunnar Brehm, Sonnenblumenweg, Germany) and a 200 V / 400 W mercury-vapor lamp (220 V / 400 W).

All specimens were photographed before the dissection of their genitalia. Images of the adults were taken using a Canon EOS 600D digital camera (Canon Inc., Ota, Tokyo, Japan). Male and female genitalia were dissected and mounted in Euparal solution, following the procedure described in Holloway et al. (1987). The genitalia slides were photographed using a digital camera attached to a Leica M205C microscope (Leica Microsystems, Wetzlar, Germany) and refined with Photoshop CS5 (Adobe Systems Inc., San Jose, CA, USA).

Most specimens examined in this study were deposited in the Systematic Entomology Laboratory, Hannam University, Daejeon, Korea (**HNSUEL**). Additional specimens examined are from the Korea National Insect Collection, Korea National Arboretum, Korea (**KNA**). Abbreviations for localities in Korea are as follows: **GG** (Gyeonggi-do), **GW** (Gangwon-do), **CB** (Chungcheongbuk-do), **DJ** (Daejeon), **GB** (Gyeongsangbuk-do), **GN** (Gyeongsangnam-do), **JN** (Jeollanam-do), and **JJ** (Jeju-do). Other abbreviations are **TL** (type locality) and **TD** (type depository).

## ﻿Systematic accounts

### ﻿Family Erebidae Leech, [1815]

**Subfamily Hypenodinae Forbes, 1954**

#### ﻿Genus Parens

Taxon classificationAnimaliaLepidopteraErebidae

Fibiger, 2011

4E106EC8-07DB-509B-9447-C4BEFC646999

##### Type species.

*Parensparaocci* Fibiger, 2011.

### ﻿Key to *Parens* species in Korea

**Table:** 

1	In female genitalia, ductus bursae coiled at terminal margin	***Parensocci***
–	In female genitalia, ductus bursae not coiled at terminal margin	***Parensfibigerina***

#### ﻿ Parens fibigerina sp. nov.

Taxon classificationAnimaliaLepidopteraErebidae

CF7A43A2-D7CF-567E-9FD0-7E8B0AB4B37D

https://zoobank.org/60C683F2-89B4-49D0-BBDA-FA1E8C3C1F84

Figs 1A , 2A, B

##### Type material.

***Holotype*** Female, Korea, Donghae-si, GW, 12.viii.2021 (BK Byun), gen. slide no. HNUSEL-6442-coll. HNUSEL.

##### Diagnosis.

This species is distinguished from *P.occi* by the shape of the signum in the corpus bursae. In *P.occi*, the cross-shaped signum in the corpus bursae, while *P.fibigerina* has a triangular signum. Additionally, the *P.fibigerina* has the forewing with a more rounded apex, a whitish-beige ground color, and a terminal margin covered with more blackish scales. The hindwing is grayish with mixed black scales. Also, the new species is distinguished from *P.chekiangi* Fibiger, 1911 by characters of the 8^th^ abdominal segment and the shape of the signum. The new species has the 8^th^ abdominal segment is 2/3 length of the posterior apophyses and is well sclerotized. The antrum is strongly sclerotized. The new species has a triangular signum in the corpus bursae, while in *P.chekiangi* has a cross-shaped signum.

##### Description.

**Figure 1.**
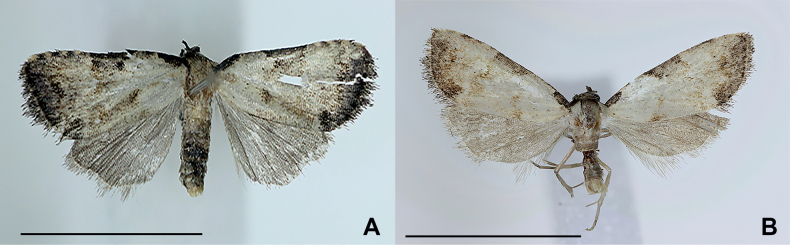
Adults of *Parens***A***P.fibigerina* sp. nov. (gen. slide no. HNUSEL_6442) **B***P.occi* (gen. slide no. HNUSEL_5931) Scale bars: 0.5 mm.

Adult (Fig. 1A). Wingspan 11 mm. Head bend down, black; antenna filiform, black; frons rounded; labial palps porrect. Thorax and abdomen with sparse scales, beige; A8–10 dark brown. Forewing ground color whitish beige, with black line from base to costal margin; base with half-round, blackish patch; apex rounded and with four yellowish blotches; antemedial and postmedial lines parallel, wavy, dark brown; spot reniform with whitish inner area and light brown outline. Hindwing ground color grayish brown, mixed black, and outline slightly curved to inner side with many cilia.

***Male genitalia*.** Unknown.

**Figure 2.**
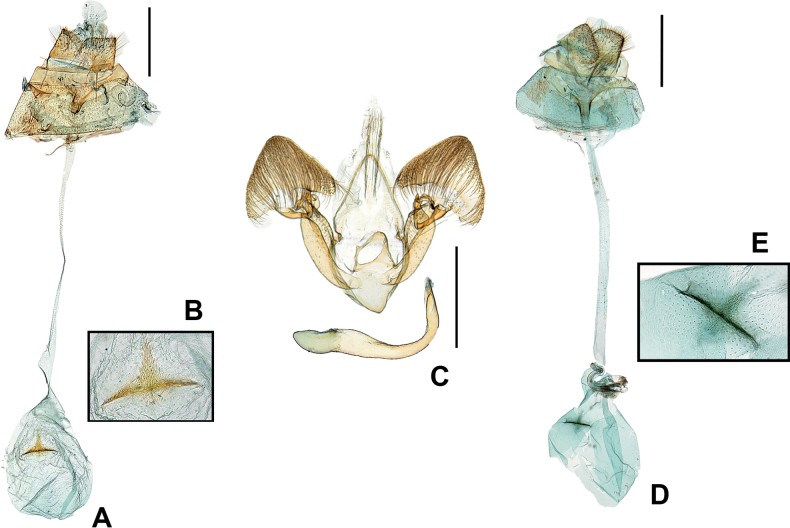
Male and female genitalia of Korean *Parens* species **A** ♀, *P.fibigerina* sp. nov., holotype (gen. slide no. HNUSEL_6442) **B** ditto, signum **C** ♂, *P.occi* (gen. slide no. HNUSEL_5931, 5937) **D** ♀, *P.occi* (gen. slide no. HNUSEL_6958) **E** ditto, signum. Scale bars: 0.1 mm.

***Female genitalia*** (Fig. 2A, B). Papillae anales normal shape, rounded apex, with many short hairs, and well sclerotized. Posterior apophyses length equal to papillae anales ; anterior apophyses extremely short. Ostium bursae placed in the median of abdomen. Antrum short, strongly sclerotized. Ductus bursae long, almost straight, narrow, membranous, and dilated at junction to corpus bursae (ca twice as broad as main tube). Corpus bursae globular and membranous, with triangular signum positioned slightly to left. Signum strongly sclerotized at base; upper side rather weakly sclerotized.

##### Distribution.

Korea (endemic).

##### Etymology.

This new species is dedicated to the memory of Michael Fibiger, a Danish entomologist, who was a renowned researcher of the tribe Micronoctuini.

#### ﻿ Parens occi

Taxon classificationAnimaliaLepidopteraErebidae

(Fibiger & Kononenko, 2008)

ADBD103F-E121-54F6-8365-1F9B70D2669A

Figs 1B , 2C–E

Micronoctua occi Fibiger & Kononenko, 2008: 52. TL: Russia, Primorye territory, Gornotaezhnoe.
Micronoctua sp.: Kononenko and Han 2007: 29.
Parens occi : Fibiger 2011: 19.
Parens occi : Lee and Byun 2022: 612.

##### Description.

**Adult (Fig. 1B).** Wingspan 10–12 mm. See Lee and Byun (2022).

***Male genitalia*** (Fig. 2C). See Lee and Byun (2022).

***Female genitalia*** (Fig. 2D, E). See Lee and Byun (2022).

##### Materials examined.

[GG] 1♂, Mt. Bongmisan, 03.xi.2008 (BW Lee, SY Park, DH Kwon), genitalia slide no. HNUSEL-5936-coll. KNAE; [GW] 1♂, Girin-myeon, 27.vi.2013 (BK Byun), genitalia slide no. HNUSEL-5931-coll. HNUSEL; 1♂, Yeongwol-gun, 26.viii.2021 (BK Byun), genitalia slide no. HNUSEL-6438-coll. HNUSEL; [CB] 2♂, Boeun-gun, 17.ix.2021 (BK Byun), genitalia slide no. HNUSEL-6309-coll. HNUSEL; Chungju-si, 10.vii.2020 (BK Byun), genitalia slide no. HNUSEL-6437-coll. HNUSEL; [DJ] 1♀, Masan-dong, 9.viii.2022 (BK Byun), genitalia slide no. HNUSEL-6958-coll. HNUSEL; [JN] 3♂, Isl. Jindo, 4.vi.2022 (BK Byun), genitalia slide no. HNUSEL-6763, 6913, 6914-coll. HNUSEL; [JJ] 1♂, Sanghyo-dong, 01.viii.2018 (BK Byun), genitalia slide no. HNUSEL-5937-coll. HNUSEL.

##### Distribution.

Korea, China, Japan, Russia (Russian Far East).

##### Remarks.

This species was reported first time from Korea by Kononenko and Han (2007).

## Supplementary Material

XML Treatment for Parens

XML Treatment for Parens fibigerina

XML Treatment for Parens occi

## ﻿Additional information

### Author ORCIDs

Ji-Young Lee https://orcid.org/0000-0001-8215-7957

Bong-Kyu Byun https://orcid.org/0000-0003-0393-6464

### Data availability

All of the data that support the findings of this study are available in the main text.

## References

[B1] Fibiger M (2011) Revision of the Micronoctuidae (Lepidoptera: Noctuoidea). Part 4, taxonomy of the subfamilies Tentaxinae and Micronoctuinae. Zootaxa 2842: 1–188. 10.11646/zootaxa.2583.1.1

[B2] Fibiger M Kononenko VS (2008) Revision of the Micronoctuidae species occurring in the Russian Far East and neighbouring countries with description of a new species (Lepidoptera, Noctuoidea). Zootaxa 1890(1): 50–58. 10.11646/zootaxa.1890.1.2

[B3] Fibiger M Han HL Kononenko VS (2011) Five new species and one new subspecies of Micronoctuidae from China, with a checklist of Chinese species, including Taiwan (Lepidoptera: Noctuoidea, Micronoctuidae). Zootaxa 2777(1): 41–53. 10.11646/zootaxa.2777.1.3

[B4] Holloway JD Bradley JD Carger DJ (1987) CIE Guides to Insects of Importance to Man 1. Lepidoptera. CAB International, London, 262 pp.

[B5] Kononenko VS Han HL (2007) Atlas genitalia of the Noctuidae in Korea (Lepidoptera). In: Park KT (Ed.) Insects of Korea Series 11. Korea National Arboretum & Center for Insect Systematics, Pocheon, 464 pp.

[B6] Lee JY Byun BK (2022) Taxonomic review of the subfamily Hypenodinae (Lepidoptera: Erebidae) from Korea. Journal of Asia-Pacific Biodiversity 15(4): 603–612. 10.1016/j.japb.2022.09.006

[B7] Zahiri R Holloway JD Kitching IJ Lafontaine JD Mutanen M Wahlberg N (2012) Molecular phylogenetics of Erebidae (Lepidoptera, Noctuoidea). Systematic Entomology 37(1): 102–124. 10.1111/j.1365-3113.2011.00607.x

